# Advances in the mechanisms, imaging characteristics and management strategies for immune checkpoint inhibitor-related pneumonitis

**DOI:** 10.3389/fimmu.2025.1656063

**Published:** 2025-09-02

**Authors:** Shengshu Li, Ziying Geng, Shuang Hong, Jianxin Zhang, Yanli Yang, Qin Wei, Xinxin Zhang, Xiaofei Zhuang, Rujie Huo, Songyan Han, Jie Wang

**Affiliations:** ^1^ Department of Respiratory Medicine, Shanxi Province Cancer Hospital/Shanxi Hospital Affiliated to Cancer Hospital, Chinese Academy of Medical Sciences/Cancer Hospital Affiliated with Shanxi Medical University, Taiyuan, China; ^2^ Department of Respiratory Medicine, University Hospital, Ludwig Maximilians University, Munich, Germany; ^3^ School of Pharmacy, Shanxi Medical University, Taiyuan, China; ^4^ Medicine Imaging Department, Shanxi Province Cancer Hospital/Shanxi Hospital Affiliated to Cancer Hospital, Chinese Academy of Medical Sciences/Cancer Hospital Affiliated with Shanxi Medical University, Taiyuan, China; ^5^ Department of Medicine, Xiamen Spacegen Co., Ltd., Xiamen, China; ^6^ College of Pulmonary and Critical Care Medicine, The Eighth Medical Center, Chinese PLA General Hospital, Beijing, China; ^7^ Department of Thoracic Oncology, Shanxi Province Cancer Hospital/Shanxi Hospital Affiliated with Cancer Hospital, Chinese Academy of Medical Sciences/Cancer Hospital Affiliated with Shanxi Medical University, Taiyuan, China; ^8^ Department of Medical Oncology, National Cancer Center/National Clinical Research Center for Cancer/Cancer Hospital, Chinese Academy of Medical Sciences and Peking Union Medical College, Beijing, China

**Keywords:** immune checkpoint inhibitor-related pneumonitis (CIP), imaging, radiomics, prediction, steroid-refractory pneumonia

## Abstract

In recent years, the introduction of immune checkpoint inhibitors (ICIs) has revolutionized the treatment landscape for malignant tumors, markedly improving survival outcomes across various cancers, such as lung cancer, esophageal cancer, and melanoma. Consequently, ICIs have become a cornerstone of first-line therapy for numerous malignancies. However, while ICIs effectively modulate immune responses to combat tumor cells, they may also trigger excessive immune activation and T-cell dysfunction, thereby leading to a spectrum of immune-related adverse events (irAEs). The organs most frequently affected by these irAEs include the skin, gastrointestinal tract, endocrine system, and lungs. Among these adverse events, the development of severe immune checkpoint inhibitor-related pneumonitis (CIP) may result in significant disability, permanent discontinuation of ICIs, and even death, with real-world incidence rates exceeding those reported in clinical trials. Early detection, precise diagnosis, and timely intervention are critical for optimizing patient outcomes. However, diagnosing CIP remains challenging because it relies heavily on high-resolution chest CT imaging and a detailed treatment history. The radiological features of CIP are often nonspecific, complicating its identification. This complexity is further exacerbated in patients receiving consolidative immunotherapy following concurrent or sequential chemoradiotherapy for stage III unresectable non-small cell lung cancer, where distinguishing between radiation pneumonitis and CIP becomes particularly difficult. To address these challenges, an increasing number of imaging experts are investigating the potential of radiomics and machine learning techniques in predicting the occurrence and assessing the prognosis of CIP. This article comprehensively reviews the pathogenesis of CIP, the predictive value of radiomics in identifying this condition and recent advancements in treatment strategies, with the aim of providing novel insights for future research and clinical management of CIP.

## 1 Introduction

In recent years, immune checkpoint inhibitors (ICIs), which target cytotoxic T-lymphocyte antigen-4 (CTLA-4), programmed cell death 1 (PD-1) and programmed cell death ligand-1 (PD-L1), have shown significant efficacy in treating various malignant tumors. ICIs meticulously modulate the activity of CD8-positive cytotoxic T lymphocytes (CD8^+^ T cells), CD4-positive helper T lymphocytes (CD4^+^ T cells), and regulatory T cells (Tregs), ultimately enhancing the antitumor immune response (1, 2). However, excessive activation of T-cell responses can disrupt the delicate balance among T-lymphocyte subsets, leading to deregulated expression of inflammatory mediators and triggering an overly aggressive immune response, potentially culminating in a spectrum of immune-related adverse events (irAEs), including but not limited to various degrees of rashes, pneumonia, hepatitis, colitis and endocrinopathies (3–5). Among these irAEs, checkpoint inhibitor-related pneumonitis(CIP) is of particular concern because of its potentially life-threatening nature. CIP manifests as the onset of respiratory system-related symptoms such as dyspnea, shortness of breath, and cough with sputum in patients receiving ICI therapy. Crucially, these symptoms are accompanied by new pulmonary infiltrates on imaging studies while excluding tumor progression, new pulmonary infections, or other conditions that could cause pulmonary changes (6). This distinctive combination of clinical and radiological features makes CIP a critical focus in the management of patients receiving ICIs.

## 2 Epidemics and risk factors

### 2.1 CIP epidemic

CIP is the most common fatal adverse reaction to PD-1/PD-L1 inhibitors (7). Compared with patients with other cancers, patients with lung cancer have a greater incidence and severity of CIP (8). Compared with monotherapy, combination immunotherapy results in more frequent CIP (9, 10). Clinical studies have reported a 3%-5% overall incidence of CIP with 1% grade≥3 cases (9, 11, 12), whereas real-world data indicate higher rates of 13%-19% (13–15).

Moey et al. (16) conducted an analysis of the WHO drug safety database and reported that the mortality rate associated with CIP was significantly higher than that reported in clinical trials, with 20.4% in lung cancer patients, potentially due to diagnostic challenges distinguishing CIP from tumor progression or infections (17). CIP may occur anytime post-ICI therapy, with a median onset of 2.8 months (range: 9 days–19.2 months) (18), rarely extending to 36 months (19). Onset occurs earlier in non-small cell lung cancer (NSCLC) patients (1.1 vs 3.1 months). Severe CIP is associated with a 61.5% mortality rate, with a median survival of 104 days (20). Consequently, for patients with a history of ICI treatment, CIP should be prioritized as a differential diagnosis if they exhibit respiratory-related symptoms or abnormal chest imaging findings.

### 2.2 Risk factors for CIP

**Figure 1:**
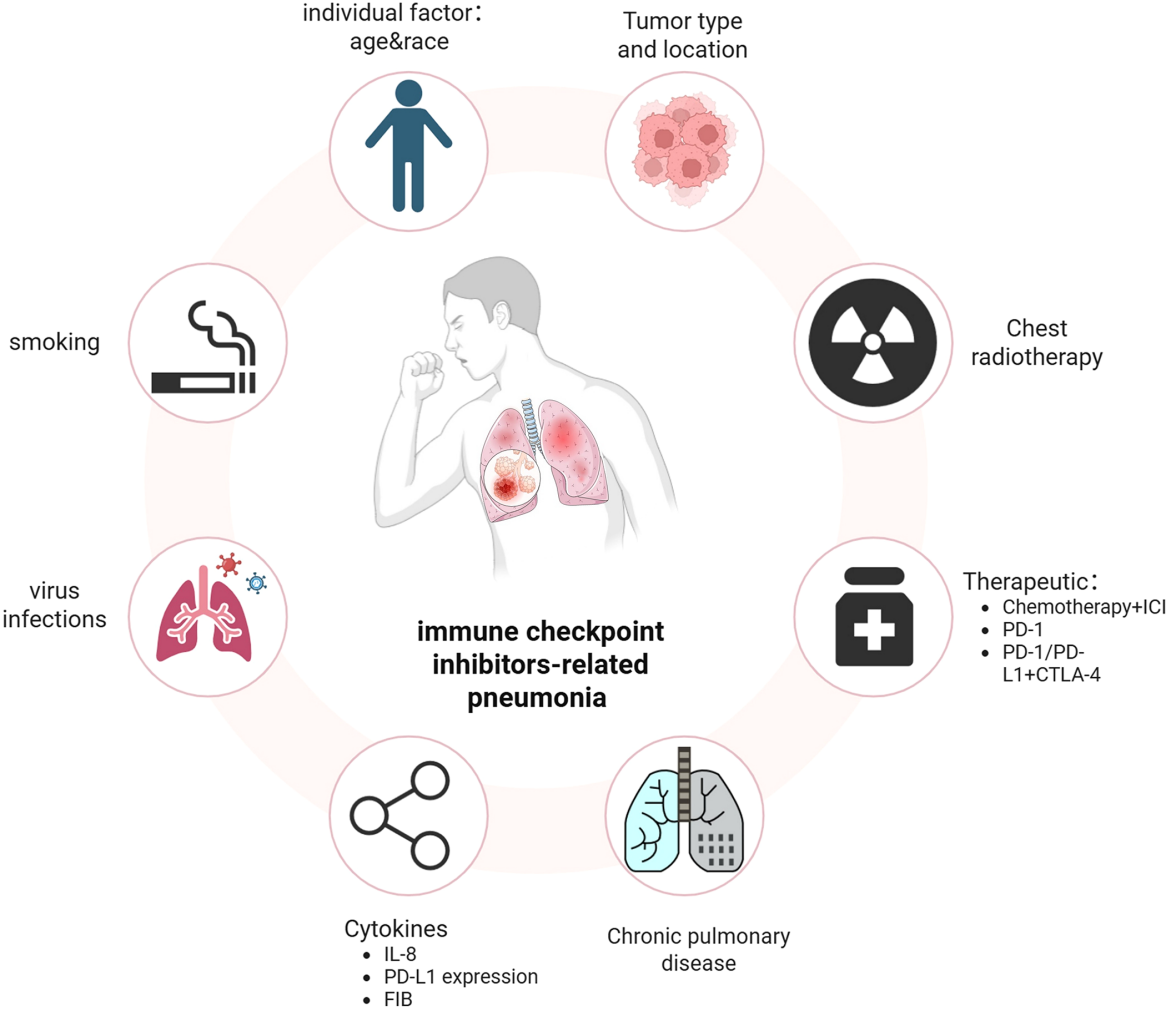
Risk factors for CIP.

Identifying the risk factors associated with CIP is crucial for facilitating early diagnosis and effective management of high-risk patients. Previous retrospective studies have reported that a high incidence of immune checkpoint inhibitor-related pneumonia (CIP) is associated with various antitumor treatments, tumor pathological types, chronic underlying lung diseases, age, smoking history, radiotherapy history, levels of certain inflammatory factors, and genetic susceptibility (**Figure 1**).

#### 2.2.1 Antineoplastic drug regimen

A meta-analysis assessing ICIs in stage III NSCLC patients revealed a significantly greater incidence of grade 2 pneumonia with anti-PD-1 therapy than with anti-PD-L1 therapy (22.7% vs 14.9%; OR=2.10, P<0.01) (21). Consistent findings were corroborated in additional studies (21). A meta-analysis revealed that the incidence of CIP was 1.6% with anti-PD-1 monotherapy vs. 6.6% with the anti-PD-1/CTLA-4 combination, indicating a greater CIP risk with dual vs. single ICI therapy (9). With the increasing prominence of perioperative neoadjuvant immunotherapy in the treatment of non-small cell lung cancer, emerging evidence indicates that the incidence of CIP among patients receiving neoadjuvant immunotherapy ranges from 3% to 25% (22). Pathological partial response (PCR) positivity rate among patients in the CIP group is significantly lower than that among patients in the non-CIP group (20.8% vs 26%) (23), while surgical intervention itself constitutes a traumatic stimulus factor. In a retrospective study, the incidence of CIP among patients who received sequential targeted drug therapy within 8 weeks following immune checkpoint inhibitor (ICI) treatment was notably greater (18.8%) than that among those who received cytotoxic chemotherapy (7.4%) and those who did not undergo any chemotherapy (5.1%) (24). Patients receiving postimmunotherapy targeted drugs developed CIP earlier than those receiving chemotherapy did (35 vs 62 days) (24). All cases of CIP were graded as having a severity of ≥3, and the condition was associated with a relatively high mortality rate (26.1%) (24). Another cohort study revealed that the median overall survival (mOS) of patients with NSCLC who received ICI therapy, such as epidermal growth factor receptor (EGFR)-TKI therapy, following tyrosine kinase inhibitor (TKI) treatment was significantly shorter than that of patients who did not receive TKIs (7.6 months vs. 18.5 months). This observed disparity may be attributed to an increased risk of immune-related adverse events (irAEs), including chemotherapy-induced pneumonitis (CIP), as well as alterations in the tumor microenvironment associated with drug resistance (25).

#### 2.2.2 Chronic lung diseases

Studies (26) have shown that, compared with noncomorbid cases, chronic obstructive pulmonary disease (COPD)/asthma in NSCLC patients is associated with an increased incidence of CIP (2.3%). Chronic interstitial lung disease (ILD) patients have a threefold greater CIP risk. UIP-type ILD patients receiving ICIs face independent acute ILD exacerbation risks (27). Conversely, isolated pleuroparenchymal fibroelastosis (PPFE) patients exhibit significantly lower CIP rates (p=0.024) and longer survival, potentially due to the lack of acute ILD exacerbation associated with PPFE, which primarily causes chronic respiratory failure (28–30).

#### 2.2.3 Site and histological type of the primary tumor

Cui et al. demonstrated a significant association between squamous cell carcinoma and the occurrence of CIP. Furthermore, obstructive pneumonia may contribute to an increased risk of CIP, as squamous cell carcinomas are predominantly central lung cancers that frequently result in obstructive pneumonia (31).

#### 2.2.4 Baseline indicators of population characteristics

A meta-analysis was conducted by Zhang et al (32). demonstrated that both race and age significantly influence the incidence of CIP. Specifically, the incidence of pneumonia is greater in Asian populations than in Western populations. Additionally, studies with a greater proportion of elderly participants revealed an increased incidence of CIP, particularly among individuals aged 65 years or older. Furthermore, a history of smoking has been identified as an independent risk factor for the development of CIP (33).

#### 2.2.5 History of radiotherapy

The KEYNOTE-001 trial revealed that the incidence of CIP was 13% in NSCLC patients receiving thoracic radiotherapy before ICI therapy versus 1% in nonradiated patients (34). Studies combining ICIs with chemoradiotherapy for locally advanced NSCLC have shown that ≥grade 2 CIP occurs in ~10% of cases (34, 35), which suggests that patients with a history of thoracic radiotherapy are at increased risk of developing CIP. Barron et al. proposed that radiotherapy-induced DNA damage, immune cell infiltration, cytokine upregulation, and collagen deposition alter the lung immune microenvironment. Thoracic radiation injury combined with hyperactivated T cells during ICI therapy may drive CIP development (36).

#### 2.2.6 Other factors

According to a study by Chao et al. (37), patients with a PD-L1 expression level of ≥50% and an IL-8 concentration of < 9.0 pg/mL presented a significantly elevated risk of developing CIP. Mao et al. (38) revealed that when the fibrinogen (FIB) level reached 3.955 g/L, the mortality rate increased by 22%, suggesting that fibrinogen, which serves as a marker of the inflammatory process, is associated with an increased risk of CIP due to its elevated levels. A retrospective study revealed that patients who tested positive for HLA-B*35 and HLA-DRB1*11 molecules presented a significantly elevated risk of developing CIP (39). It is currently recognized that in autoimmune diseases, the expression of HLA-B*35 is linked to an increased risk of severe primary pulmonary hypertension in scleroderma patients, as well as to nephritis accompanied by marked leukocytosis and high-risk juvenile idiopathic arthritis. Furthermore, HLA-DRB1*11 expression has been associated with the development of systemic sclerosis (40–42). The human leukocyte antigen (HLA) complex is crucial for the efficacy and regulation of T-cell-mediated immune responses. It specifically functions by presenting peptides derived from tumor antigens to T-cell receptors on effector T cells (43). The proinflammatory effects and endoplasmic reticulum stress (ERS) induced by the HLA-B*35 allele, combined with the Th2-promoting capability of DRB1*11, may explain the increased incidence of immune-related pneumonitis (IRP) in cancer patients receiving PD-1/PD-L1 immune checkpoint inhibitor therapy (42, 44). A previous study (45) reported that the incidence of CIP follows a seasonal pattern, with a higher prevalence observed during the winter months. These findings suggest a link between ICI treatment, viral infections, and CIP, indicating that seasonal viruses may contribute to CIP development. Additionally, the literature suggests a potential association between cytomegalovirus (CMV) infection and the development of CIP. In this study, serological samples from 29 patients diagnosed with grade 3–4 CIP were analyzed, and CMV pp65 antigen was positive in 28 of these patients (46).

## 3 Progress on the mechanisms of CIP

**Figure 2:**
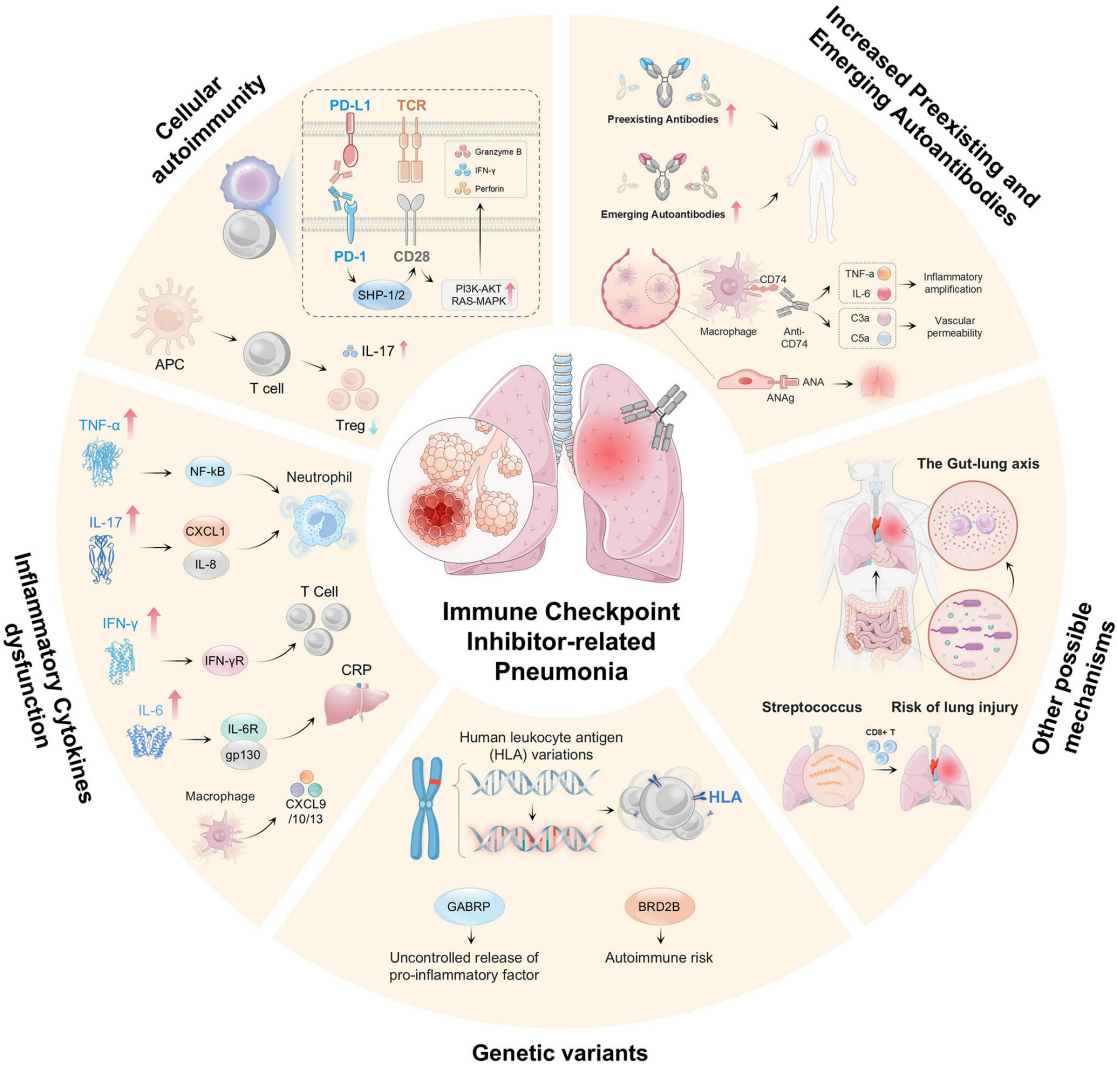
Shows that CIP development involves multiple mechanisms and factors. These include dysregulation of the PD-1/PD-L1 and CTLA-4 pathways, which causes autoimmune dysfunction; excessive T-cell activation and cytotoxicity with impaired Treg function; imbalance in cytokine networks (e.g., elevated TNF-α, IL-6, IL-17, and IFN-γ) and proinflammatory mediators (e.g., CXCL1, IL-8, and CXCL9/10/13), which activate neutrophils and T cells; genetic variations, which affect immune responses (e.g., HLA polymorphisms, GABRP, and BRD2B); autoantibodies, which amplify inflammation via complement activation (e.g., C3a/C5a); gut–lung axis imbalance (e.g., altered microbiota), which increases the risk of lung injury; and infections (e.g., streptococcal), which trigger autoimmune reactions.

The molecular mechanism of CIP involves a complex interplay of multiple factors. Although the precise molecular mechanisms underlying this condition remain incompletely understood, several key processes are believed to contribute to the onset of pneumonitis during ICI treatment (**Figure 2**). Emerging scientific evidence suggests the involvement of diverse pathways in the pathogenesis of CIP, which are delineated below.

### 3.1 T-cell dysfunction

PD-1/PD-L1 inhibitors can augment the antitumor efficacy of T cells (47, 48). Accumulating evidence indicates that the upregulation of T cells may play a role in the pathogenesis of CIP (4, 49). The increased activity of these targeted T cells can lead to the attack of cross-reactive antigens shared between tumors and normal lung tissue, resulting in off-target toxicity.

Hiroyuki and colleagues reported the presence of T-cell-predominant lymphocytosis in the BALF of patients with CIP (50). Notably, the proportions of CD8^+^ T cells expressing immune checkpoint proteins, a hallmark of tumor-infiltrating T lymphocytes (TILs), are greater in patients with CIP than in those with other immune-related adverse events (irAEs) (51–53). Naidoo et al. proposed increased infiltration of highly proliferative CD8^+^ T cells in the lung biopsy tissue of NSCLC patients who developed chronic bronchiolitis obliterans organizing pneumonia subsequent to nivolumab therapy (54). Subudhi et al. demonstrated a significant correlation between the abundance of CD8^+^ T-cell clones in the peripheral blood and the incidence of irAEs (P=0.01), particularly grade 2–3 irAEs (P<0.0001), among patients treated with ipilimumab, a CTLA-4 inhibitor (55, 56). Suresh and colleagues reported a significant increase in CD4^+^ T cells in the BAL samples of CIP patients, primarily those with NSCLC, following treatment with anti-PD-1/PD-L1 inhibitors (57). These findings support a lymphocyte-driven hyperimmune response.

In addition, the expression levels of CTLA-4 and PD-1 on Treg cells in CIP patients were significantly decreased, suggesting an attenuated Treg suppressive phenotype (57, 58). When regulated by ICIs, CTLA-4, a key molecule in Treg cell function, can alter the suppressive capabilities of Treg cells within the tumor immune microenvironment. This alteration may lead to the development of CIP by relieving the immunosuppressive effects of Treg cells (2, 59). Moreover, deficiency of CTLA-4, in addition to PD-1, may further compromise the ability of Treg cells to control the proinflammatory responses of conventional T cells and macrophages, thereby exacerbating the unchecked immune dysregulation observed in CIP (57).

T-cell activation and infiltration within the lung tissue of CIP patients signify enhanced antitumor immunity. However, this heightened immune activity poses a risk of normal tissue damage due to overly aggressive immune reactions (60, 61), which support the hypothesis that ICI-related pneumonitis may be induced when ICI-activated T cells recognize self-peptides or epitopes that are shared between the tumor and the host (62). This hyperresponsive immune state, triggered by T-cell activation and infiltration, may lead to the misidentification of self-antigens in lung tissue, thereby triggering an autoimmune reaction (63).

### 3.2 Increased preexisting and emerging autoantibodies

An increasing number of studies indicate that the occurrence of CIP may be linked to elevated levels of both preexisting and newly emerging autoantibodies within the human immune system (58, 64). These autoantibodies, which may be present at low concentrations before the initiation of ICI therapy or generated *de novo* during treatment, are believed to contribute to the pathogenesis of certain nonpulmonary irAEs (58).

PD-1/PD-L1-targeted therapy has been shown to induce Treg dysfunction and foster the generation of pathological autoantibodies, as evidenced in both PD-1-knockout mice and patients undergoing such treatment (65, 66). Adverse effects such as pneumonitis, arthralgia, vitiligo, and hypothyroidism are frequently observed in patients receiving PD-1/PD-L1 inhibitor treatment (67, 68). Additionally, Toi et al. demonstrated that NSCLC patients have preexisting autoantibodies, such as rheumatoid factor (69).

Klocke et al. investigated the role of CTLA-4 by ablating CTLA-4 expression in adult mice and compared the resulting autoimmunity with that observed in congenital CTLA-4 deficiency. They identified autoimmune disease phenotypes, including pneumonitis, accompanied by organ-specific autoantibodies (70). In a retrospective study of 22 patients with CTLA-4 insufficiency and COVID-19, Ochoa et al. examined autoantibodies against type 1 IFNs at baseline. They reported that most patients with CTLA-4 insufficiency and COVID-19 had nonsevere disease and lacked autoantibodies against type 1 Interferons (71). These findings suggest a potential explanation for the risk of developing autoimmune complications in cancer patients during treatment with the CTLA4-blocking checkpoint inhibitor ipilimumab (72).

Emerging evidence implicates multiple distinct autoantibodies in the development of CIP. Notably, CD74, an autoantibody-active protein, functions as an intracellular chaperone for major histocompatibility complex class II (MHC-II) and can stimulate the release of inflammatory mediators. Although primarily intracellular, CD74 is also expressed on the cell membrane of immune cells, including macrophages (73). It serves as a high-affinity receptor for macrophage inhibitory factors, thereby inducing inflammatory mediator release and cell proliferation (74, 75). In normal human lung tissue, CD74 is expressed at modest levels; however, its expression is dramatically increased in lung tissues affected by ICI-induced pneumonitis (76). A large-scale screening study of ICI-treated patients revealed that serum levels of anti-CD74 antibodies were elevated in patients with CIP compared with their pretreatment levels (77). Salahaldin A. Tahir et al. conducted a high-throughput serological analysis of recombinant cDNA expression to investigate autoantibodies in patients receiving ICIs. They reported a significant median 1.34-fold increase in anti-CD74 antibody levels post-ICI treatment in patients with CIP, whereas no significant changes were detected in a comparison group of 20 patients without pneumonitis, suggesting a pathogenic role for CD74 autoantibodies in the development of pneumonitis (78). These findings indicate the pathogenic involvement of CD74 and its autoantibodies in the development of CIP.

### 3.3 Inflammatory cytokine imbalance

The administration of ICIs can activate T cells, resulting in excessive release of cytokines and potent proinflammatory responses, which in turn promote the development of various adverse events, including CIP. Elevated levels of various cytokines have been observed in patients experiencing irAEs following ICI treatment, highlighting significant changes in cytokine profiles (79). Moreover, certain cytokines have shown promise as predictive biomarkers for irAEs, offering promising avenues for early detection and management strategies.

Tumor necrosis factor-α (TNF-α), interleukin-6 (IL-6) and interferon-γ (IFN-γ) collectively contribute to tissue damage and exacerbate the immune-mediated lung injury characteristic of CIP (63, 80, 81). Dysregulated cytokine production disrupts immune homeostasis by initiating inflammatory cascades, which recruit neutrophils and macrophages to injury sites and suppress anti-inflammatory counterregulation, ultimately worsening CIP severity (82–84). Mechanistically, Lin et al. demonstrated that the infiltration of Th2 cells in the BALF of CIP patients drives the overproduction of interleukin clusters (IL-4, IL-5, IL-6, IL-9, IL-10, and IL-13), thereby creating a self-sustaining inflammatory loop (85).

Similarly, other studies have also identified specific cytokines associated with the development of irAEs, including pneumonitis. Lim’s team, who studied melanoma patients receiving ICIs, identified 11 plasma cytokines (including granulocyte–colony-stimulating factor [G-CSF], granulocyte–macrophage colony–stimulating factor [GM–CSF], and IL–13) that dynamically increase before or during the onset of high-grade irAEs (86). Notably, NSCLC studies have revealed both similarities and divergences in cytokine patterns: while elevations in IL-1β and IL-8 align with findings in melanoma, decreased G-CSF has emerged as a unique predictor in lung cancer cohorts (87). This discrepancy underscores microenvironment-driven variations in the cytokine network. Furthermore, a study of 204 NSCLC patients with or without PD-L1 inhibitor monotherapy (43 of whom developed irAEs) revealed a proinflammatory increase in IL-1β and elevated levels of IL-5, IL-8, IL-10, IL-12p70, and granzyme A, along with decreased G-CSF, as predictors of irAEs, including pneumonitis (86). Receptors for IL-8 are present on neutrophils, Tregs, monocytes, and NK cells, indicating their potential involvement in the complex biology of CIP (49, 88). However, the role of IL-5 and IL-10 in lung injury associated with pneumonitis remains uncertain.

IL-6 has garnered significant attention in CIP research because of its potential role in its pathogenesis. One study reported elevated IL-6 levels in CIP patients compared with those at baseline, whereas another examination of BALF cytokines in 12 CIP patients revealed significantly higher IL-6 levels than in controls (89, 90). However, it is important to note that IL-6 elevation is not universal in the BALF of CIP patients (57). Despite this variability, tocilizumab, an IL-6 inhibitor, has shown efficacy in treating steroid-refractory CIP in a single-center study (91). C-reactive protein (CRP), an acute-phase protein secreted by liver cells in response to inflammatory cytokines such as IL-6 and TNF-α, is also relevant. Given the observed elevated IL-6 levels in some CIP patients, it is unsurprising that CRP levels are increased in NSCLC patients who develop CIP postatezolizumab treatment compared with baseline (92, 93). These findings suggest that CIP development may be attributed to excessive immune system activation induced by both CRP and IL-6, along with their potent proinflammatory properties (81).

IL-17, a cytokine with diverse functions, plays crucial roles in autoimmune diseases and inflammation. Its abnormal expression has been implicated in the pathology of various lung diseases, including asthma, pneumonia, and pulmonary fibrosis (94). Lou et al. demonstrated a significant increase in serum IL-17 levels in NSCLC patients who developed CIP following ICI treatment (95). This phenomenon may be attributed to the disruption of immune tolerance and enhanced T-cell activation resulting from PD-1 and PD-L1 blockade (96, 97). A separate analysis of serum and BALF from 13 NSCLC patients with CIP post-PD-1/PD-L1 therapy revealed elevated levels of both IL-17A and IL-35 in these compartments. Furthermore, serum IL-17A levels were found to be positively correlated with the Th17 cell subtype (98). Given that IL-17A is implicated in other autoimmune disorders, acute lung injury, and lung fibrosis, it is plausible that it also contributes to the pathogenesis of CIP (49, 99, 100).

C-X-C chemokines (CXCLs) are pivotal in regulating the differentiation of primary T cells into Th1 cells and facilitating the migration of immune cells to tumor sites through interactions with molecules such as CXCL9, CXCL10, CXCL11, and CXCL13 (101, 102). By assessing 40 cytokines in plasma, Shaheen Khan and colleagues reported significant upregulation of various cytokines, particularly CXCL9, 10, 11, and 13, following ICI treatment, which was closely linked to the onset of irAEs (103).

Despite the variability in underlying tumor histology, host factors, and disease profiles among patients, emerging evidence suggests that cytokine dysregulation is involved in the pathogenesis of CIP. This dysregulation leads to the release of proinflammatory cytokines and the infiltration of immune cells into the lung parenchyma, thereby exacerbating tissue damage and contributing to the development of pneumonitis (81).

### 3.4 Genetic variants

Given the widespread belief that the development of irAEs is linked to autoimmunity, genetic variations have emerged as a potential contributing factor to this phenomenon. Notably, human leukocyte antigen (HLA) variations, which are crucial at the immune cell interface, have garnered significant attention in recent research. In a cohort of 256 patients undergoing PD-1/PD-L1 treatment, including 29 CIP patients, HLA typing revealed a strong correlation between the incidence of CIP and the germline expression of HLA-B allele 35 and HLA-DRB1 allele 11 (39). These alleles have also been implicated in other autoimmune disorders, further underscoring the potential significance of genetic factors in the pathogenesis of CIP (104). Furthermore, specific genetic polymorphisms have been associated with susceptibility to CIP. These include polymorphisms in genes related to immune regulation and inflammation, such as those encoding gamma-aminobutyric acid type A receptor subunit pi (GABRP), desmocollin, and bromodomain adjacent to zinc finger domain 2B (BRD2B). However, further research is necessary to elucidate their precise roles in the development of CIP (82, 105). Collectively, these findings suggest that genetic variations are likely to contribute to the dysregulation underlying CIP.

### 3.5 Other possible mechanisms

Hakozaki et al. highlighted disparities in the gut microbiome between advanced NSCLC patients experiencing low- and high-grade irAEs (106). This has prompted a growing focus on the ‘gut–lung axis’, a concept describing the interaction between the gut and respiratory microbiomes and its potential impact on immune responses (107). Although research on respiratory microbiomes in the context of cancer immunotherapy is relatively less extensive than that on gut microbiomes, studies have indicated that anti-PD-1 treatment can modulate the diversity and abundance of specific microbiome populations (108, 109). Notably, Zhang et al. discovered that certain respiratory microbes, particularly enriched Streptococcus, might potentiate antitumor immune responses by enhancing antigen presentation and effector T-cell function, thereby potentially increasing the incidence of irAEs, including CIP (110).

Hsu et al. demonstrated that PD-1-expressing NK cells can mediate immunosuppression in tumor microenvironments by interacting with PD-L1-positive tumor cells. PD-1/PD-L1 blockade can release the cytotoxic potential of NK cells against tumors by increasing PD-1-induced immune inhibition. However, when ICI treatment triggers inflammation in nonmalignant lung tissue via diverse non-NK cell-mediated pathways, activated NK cells can directly eliminate infected cells and secrete proinflammatory cytokines. Consequently, NK cell activation mediated by PD-1/PD-L1 blockade might exacerbate inflammation and contribute to damage in normal lung tissues (111).

## 4 Imaging manifestations of CIP

In a previous retrospective study, Park et al. (112) initially identified common imaging features of CIP, including ground-glass opacity (GGO), consolidation, reticular density, and traction bronchiectasis. In Pozzessere’s study (113), CIP was categorized into eight distinct types: organizing pneumonia (OP), nonspecific interstitial pneumonia (NSIP), hypersensitivity pneumonia (HP), acute interstitial pneumonia/diffuse alveolar damage (AIP/DAD), bronchiolitis, nodular or mass-like lesions, the unclassifiable type, and sarcoidosis-like manifestations. The predominant distribution pattern of lung injury induced by ICIs is bilateral, involving multiple lobes and segments. However, unilateral or single-lobe lesions may also occur. The lower lobes are most frequently affected. In recurrent cases, the imaging distribution may remain consistent or exhibit migratory characteristics (45, 114).

### 4.1 Classical imaging manifestations of CIP

The 2021 Fleischner guidelines categorized the CT imaging patterns associated with drug-induced pneumonia into five distinct types (115), four of which can be observed in patients receiving immunotherapy.

#### 4.1.1 Organizing pneumonia (OP)

**Figure 3:**
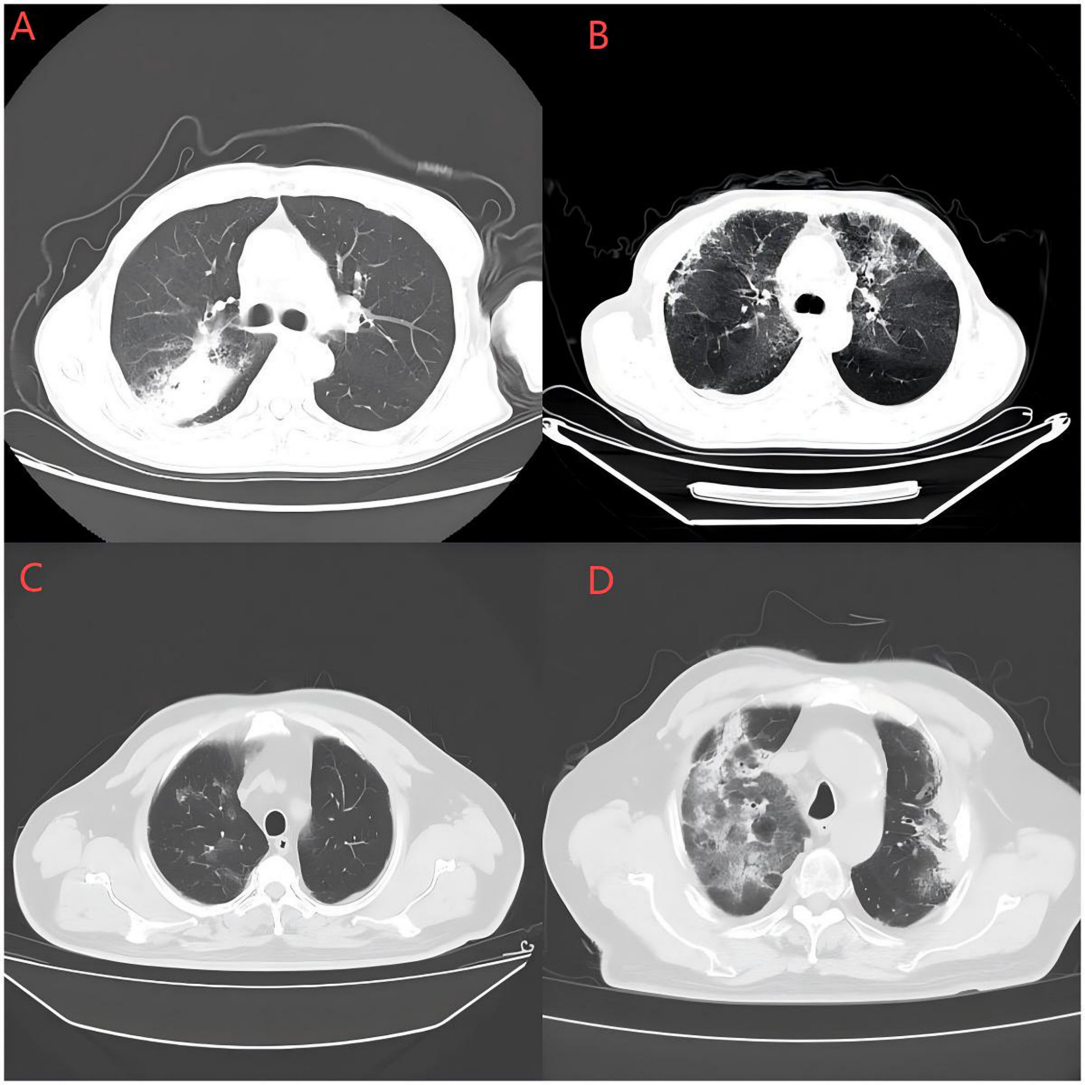
Classic CT imaging of CIP. **(A)** Organizing pneumonia (OP); **(B)** Nonspecific interstitial pneumonia (NSIP); **(C)** Hypersensitivity pneumonia (HP); **(D)** Acute interstitial pneumonia/diffuse alveolar damage (AIP/DAD).

OP is characterized by multiple patchy areas of increased density, which are typically distributed around bronchovascular bundles and/or in the peripheral lung fields, and may be associated with a reverse halo sign (**Figure 3A**). Histologically, it is characterized by granulation tissue filling the alveolar ducts and surrounding alveolar spaces, accompanied by inflammatory infiltration of the lung parenchyma (116). Bronchoalveolar lavage revealed a decreased CD4/CD8 T-cell ratio, with an increase of 20% to 40% in activated T lymphocytes (117).

#### 4.1.2 Nonspecific interstitial pneumonia (NSIP)

NSIP presents with imaging features ranging from patchy ground–glass opacities to irregular reticular shadows, accompanied by destruction of the lung lobe structure and traction bronchiectasis, with or without consolidation (**Figure 3B**). Lesions typically exhibit bilateral and symmetrical distributions, predominantly involving the lower lobes of the lungs. A distinguishing feature of NSIP compared with OP is the relative sparing of subpleural regions (116). Histologically, NSIP is characterized by lymphocytic and plasma cell infiltration, as well as uniform thickening of the alveolar walls (118).

#### 4.1.3 Hypersensitivity pneumonia (HP)

The HP pattern comprises indistinct centrilobular nodules, ground-glass opacities in both lungs, and large patchy or lobar regions exhibiting mosaic perfusion (**Figure 3C**). The CTCAE grade for the HP pattern tends to be relatively low, typically ranging from grade 1 to 2 (114). Histologically, it is distinguished by diffuse lymphocytic and plasmacytic infiltration surrounding the bronchi, as well as loose, nonnecrotizing granuloma formation (119).

#### 4.1.4 Diffuse alveolar damage (DAD)

DAD, also referred to as AIP or acute respiratory distress syndrome (ARDS), is characterized by extensive ground–glass opacities with consolidation in both lungs during the exudative phase. The organizing and fibrotic stages are characterized by traction bronchiectasis, whereas in the late stage, there is a notable reduction in lung volume (**Figure 3D**). Additionally, DAD may present as thickening of the interlobular and intralobular septa, forming a “crazy paving” pattern (120). The clinical manifestations and pulmonary injury associated with the AIP pattern tend to be severe, typically corresponding to a CTCAE grade of 3 or higher (114). Histologically, DAD is characterized by diffuse alveolar damage and pulmonary edema (120).

### 4.2 Radiation recall pneumonitis (RRP)

**Figure 4:**
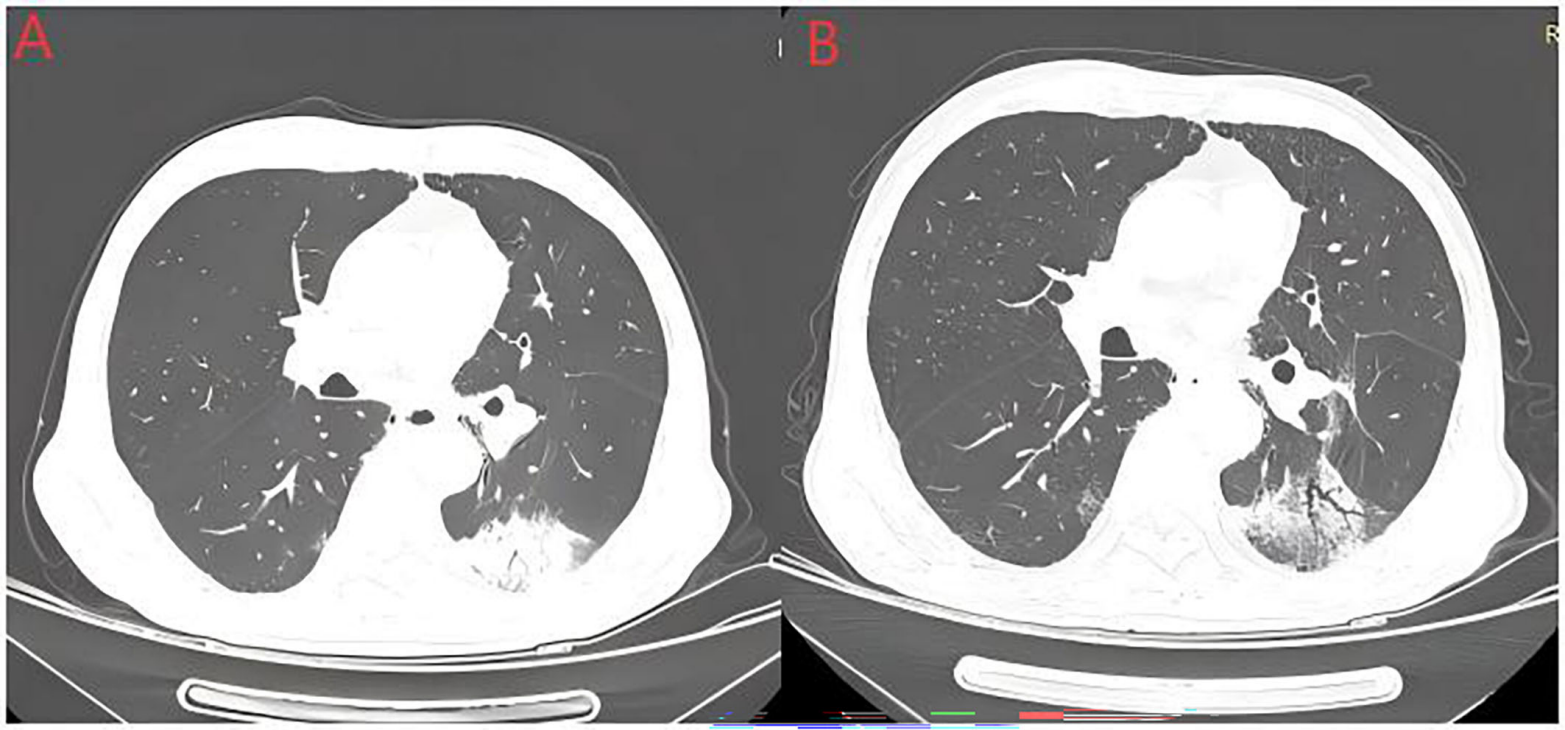
Radiation recall pneumonitis (RRP). The above image shows an elderly man with a history of smoking who was diagnosed with stage IIIB squamous cell carcinoma. He received 4 cycles of albumin-bound paclitaxel combined with carboplatin treatment, followed by radical radiotherapy. Image **(A)** shows the occurrence of pneumonia after one cycle of tislelizumab treatment following sequential chemoradiotherapy. Image **(B)** shows partial absorption of pneumonia in the left lower lobe after 2 weeks of methylprednisolone therapy.

In addition to the classic manifestations of CIP, radiation recall pneumonitis (RRP) has attracted increasing interest from oncologists and radiation therapists in recent years. The incidence of RRP has increased since immunotherapy, which is used as a consolidation treatment following chemoradiotherapy for locally advanced NSCLC, was promoted clinically. Some studies report that the incidence of RRP can reach 18.8%, with a median onset time of 450 days post-radiotherapy (116). RRP is characterized by CT-detected pneumonia outside the typical radiation pneumonitis window (4–12 weeks post-RT) and is localized to the irradiated area (**Figures 4A, B**). Imaging features include patchy ground-glass opacity/consolidation (120). Pathologically, it is characterized by mucosal congestion, leukocyte infiltration, alveolitis, type II alveolar cell hyperplasia, and fibrosis (121). ICI-induced RRP results in radiation field-aligned consolidation/ground-glass opacity with clear lesion-normal tissue borders. ICI-induced RRP mechanisms may involve overactivation of T lymphocytes post-ICI binding to irradiated lung tissue, triggering hypersensitivity; radiation-induced endothelial damage, increasing vascular permeability and localized ICI accumulation in irradiated areas; and synergistic damage to alveolar epithelial cells caused by both therapies (122, 123).

### 4.3 Other rare imaging modalities

Bronchiolitis has been reported exclusively in case studies. Histologically, bronchiolitis is obliterative and characterized by centripetal fibrosis of the submucosa and peribronchial tissues of the terminal and respiratory bronchioles (124). CT findings include thickened bronchial walls and diffuse tree-in-bud opacities in the peripheral lung zones, often accompanied by bronchiectasis or sharply demarcated mosaic attenuation patterns of the lung parenchyma, which are more pronounced during expiration (125). SLR represents a drug-induced multisystem granulomatous reaction that is histologically characterized by noncaseating epithelioid granulomas with multinucleated giant cells at the center and surrounding scattered lymphocytes (126). It occurs in approximately 5% to 7% of patients receiving nivolumab therapy (127). Typical HRCT features include miliary nodules predominantly distributed in the upper and middle lung zones along the lymphatic vessels, which are associated with mediastinal and bilateral hilar lymphadenopathy (128). Most SLR cases require no treatment owing to minimal symptoms. ICI-induced AEP is rare and presents as bilateral ground–glass opacities, consolidation, septal thickening, and pleural effusion on CT. Diagnosis requires elevated eosinophils in the blood and bronchoalveolar fluid. Monitoring blood eosinophils during ICI therapy aids early detection of CIP (129, 130).

Imaging exams are crucial for diagnosing CIP, with manifestations linked to disease progression, hormone therapy response, and prognosis. Studies have shown that organizing pneumonia (OP) is the most common CT pattern in CIP patients (114). Clinically, the severity of CIP follows the order of DAD/AIP > NSIP/HP > OP (131). Capaccione et al. (132) demonstrated through multimodal imaging analysis that the imaging characteristics of CIP patients are significantly associated with the type and duration of immunotherapy. Specifically, CIP linked to anti-PD-1/PD-L1 therapy predominantly exhibits an OP pattern, whereas CIP associated with anti-CTLA-4 therapy typically manifests as an NSIP pattern. Research indicates that individuals in CIP stages G1–2 have higher survival rates than those in G3–4. Hormone therapy benefits HP/OP-like patients, whereas DAD-like patterns show rapid progression and poor treatment response (33, 115).

### 4.4 CIP with infection

Clinically, patients with CIP often exhibit no specific symptoms, and approximately one-third of them may remain asymptomatic. Therefore, differential diagnosis is essential to exclude other potential conditions, such as pulmonary infection, tumor pseudoprogression, radiation-induced lung injury, and pulmonary edema. Among them, opportunistic pulmonary infections, including tuberculosis (TB), aspergillosis, cytomegalovirus pneumonia (CMVP), and *Pneumocystis jirovecii* pneumonia (PJP), present significant diagnostic challenges and constitute important differential diagnoses (133–135). A meta-analysis revealed that patients treated with PD-1/PD-L1 inhibitors are not only at risk of developing various types of immune-related pneumonitis but also exhibit an increased incidence of infectious pneumonia compared with patients in the chemotherapy or placebo groups (136). The presence of symptoms such as fever, expectoration, and elevated blood counts may indicate infection in patients. Obstructive pneumonia represents a common type of pulmonary infection among lung cancer patients, with bacterial pathogens being the most prevalent cause. *P. jirovecii* infection may result in bilateral ground–glass opacities and hypoxemia, whereas viral infections can also cause diffuse pulmonary involvement. Furthermore, several case reports have documented fungal infections, fungal airway disease, and active pulmonary tuberculosis in patients receiving ICI therapy (137–139). Pulmonary infection and CIP may present with overlapping imaging features, making differential diagnosis challenging. Therefore, it is essential to integrate findings from sputum microbiology and serum biomarkers, including Legionella antibodies, Mycoplasma antibodies, (1, 3)-β-D-glucans, Aspergillus galactomannan, Cryptococcus capsular polysaccharide antigen, and interferon-γ release assays, to facilitate accurate differentiation (140). In patients who develop immune checkpoint inhibitor-associated pneumonitis (CIP), particularly those with grade 2 or higher CIP, bronchoscopy and/or bronchoalveolar lavage (BAL) are recommended as initial diagnostic procedures to exclude infectious etiologies. When concurrent infection is suspected, prompt initiation of empirical anti-infective therapy is also warranted (120). In cases where CIP and infectious pneumonia cannot be definitively differentiated, occur concurrently, or are followed by secondary infection, empirical antibiotic therapy should be initiated following appropriate diagnostic evaluation, while simultaneous efforts are made to identify the etiological agent. Moreover, throughout the management of CIP, continuous monitoring is needed for opportunistic infections that may arise as a consequence of immunosuppressive therapy (141).

## 5 Radiomics in CIP: from basic imaging features to advanced predictive modeling

Early studies focused on the basic imaging features of CIP. With the development of radiomics, subsequent studies explored the links among imaging findings, histopathology, treatment response, and the potential of radiomics for assessing CIP risk, diagnosis, and prognosis. Radiomics extracts high-throughput data from medical images via computational algorithms, transforming qualitative metrics into quantitative metrics. It includes traditional and deep learning-based types. Common feature selection methods in traditional radiomics include least absolute shrinkage and selection operator (LASSO) (142), principal component analysis (PCA) (143), and minimum redundancy maximum relevance (MRMR) (144). However, traditional radiomic approaches may overlook higher-level features during feature extraction. Deep learning-based radiomics uses CNNs and transformers for forward propagation to generate predictive outcomes through data transformation. Backpropagation optimizes network parameters via gradient calculations from prediction errors. This interaction enables automatic feature extraction and advanced radiomic analysis. The CNN remains the classical architecture widely applied in medical imaging, with GoogLeNet/Inception, ResNet, VGGNet, and DenseNet being prominent models in lung cancer radiomics (145).

### 5.1 Radiomics-driven CIP risk stratification

In 2019, Colen et al. (146) pioneered a radiomic model for predicting CIP in 32 patients. Using 3D Slicer for CT image segmentation, they extracted 1,860 texture features on the basis of histograms and the GLCM. The mRMR method selected predictive features, achieving 100% accuracy (p = 0.0033). However, the small sample size limits the generalizability of the results. Building on Cole’s work, Zhang et al. (147) increased the robustness of the NSCLC prediction model by integrating greyscale-dependent matrix radiomic features. Features were selected via combined MRMR and RFE methods, with added model evaluation techniques. The ICC consistency assessment ensured reliability. Ultimately, the model constructed using the RS, clinical, and SF features demonstrated excellent performance in terms of ROC curves, calibration curves, and DCA. This advancement enables physicians to predict the likelihood of CIP in NSCLC patients with greater speed and accuracy.

Tan et al. (148) developed a multimodal deep learning model using a 3D ResNet18 architecture to predict chemotherapy-induced pneumonia in lung cancer patients. The model integrated nine clinical and radiomic features through 18 convolutional layers and fully connected layers. Compared with two-stage transfer learning with contrastive learning, it achieves an AUC of 0.918 via fivefold cross-validation. Similarly, Cheng et al. (149) developed a multimodal nomogram integrating clinical and deep imaging radiomics to predict CIP risk in 141 lung cancer patients. They optimized ResNet-50-V2 with a feature pyramid network (FPN), showing that the multimodal model outperformed the radiomic-only and clinical-only models, achieving AUCs of 0.910 vs 0.871 vs 0.778 (training set) and 0.900 vs 0.856 vs 0.869 (test set), respectively.

Chen et al. (150) constructed a radiomic prediction vector for lung cancer immunotherapy (LCI-RPV) on the basis of radiomic tumor features from enhanced chest CT scans, CD274 counts in NSCLC patients treated with anti-PD-1/PD-L1 therapy, and RNA expression levels of the PD-L1 protein-coding gene as the response variable. Subgroup analysis revealed that the LCI-RPV could predict CIP in PD-L1 inhibitor patients, with an AUC of 0.74 (95% CI: 0.53–0.95). Thomas et al. pioneered functional lung radiomics in locally advanced NSCLC, assessing its role in pneumonia risk stratification for combined radiation and ICI therapy. Baseline COPD significantly increased pneumonia risk (HR 4.59). COPD was the primary predictor, with a c-index of 0.69 (0.59–0.80) (151).

### 5.2 Identification of CIPs

The incidence of RP and CIP is greater than that of other types of treatment-related pneumonitis in NSCLC patients. RP is dose dependent with radiation field confinement, whereas CIP presents bilateral multilobe involvement without dose correlation (120, 152, 153). Concurrent RT-ICI-induced pneumonia combines the features of CIP and RP (11, 154) rather than simple superposition. In the PEMBRO-RT trial and PACIFIC study, the incidence of pneumonia in NSCLC patients receiving radiotherapy (RT) and ICI was reported to be as high as 26% to 33.9% (95, 155). Differentiating CIP from RP is crucial for clinical management, guiding steroid dosage and ICI rechallenge or discontinuation (156).

Chen et al. (157) analyzed CT data from 82 NSCLC patients with pneumonia (immunotherapy/radiotherapy/combined therapy). They built a radiomic model using LASSO-selected features and grid search optimization, achieving a training set AUC of 0.76 for distinguishing ICI- and RT-induced pneumonia. Cheng et al. (158) analyzed CT images and clinical data from 73 patients with pneumonia related to ICIs, radiotherapy or combined therapy. Three CT texture features (intensity histogram, GLCM-based, and Bow) were extracted, with the Bow showing the best cross-validation performance (AUC 0.937). Logistic regression, random forest, and linear SVM models underwent 10-fold validation in ICI/radiotherapy-only patients. Testing in combined therapy patients yielded AUCs of 0.765, 0.848, and 0.937, respectively. The optimal model achieved an AUC of 0.896 in the combined treatment cohort, outperforming prior studies. QIU et al. (159) developed a CT radiomic model that integrates radiomic, clinical, and radiological factors to differentiate CIP from RP, with excellent performance (AUC 0.953 and 0.947). Bilateral CT changes were more likely in patients with ICI-induced pneumonia than in those with RP (p<0.001). CIP patients had less well-defined boundaries (p=0.001), which aligns with other studies (157). The radiomic nomogram outperformed the Rad-score in distinguishing CIP from RP.

Wang et al. (160) developed a CT-based radiomic model for RP and CIP patients. They built two models (random forest and linear discriminant classifiers), achieving test cohort AUCs of 0.851 and 0.842, respectively, with 83% accuracy. Additionally, this study identified four highly discriminative features, including one first-order feature, one gray-level run-length matrix (GLRLM)-based feature, one gray-level co-occurrence matrix (GLCM)-based feature, and one neighborhood gray-tone difference matrix (NGTDM)-based feature. Previous studies (161, 162) have shown that multifrequency CT decomposition effectively decodes the underlying phenotypic differences between CIP and RP in patients undergoing RT and ICI therapy.

The De Ruysscher team published a study in 2021 at the European Society for Medical Oncology (ESMO), which distinguished CIP from other causes of pneumonia (163). They extracted 837 radiomic features and used recursive feature elimination (RFE) to select the 42 most correlated features, and the areas under the ROC curves of the clinical, radiomic, and combined models for predicting ICI-R pneumonitis were 0.99, 0.65, and 0.99, respectively.

Early identification and diagnosis of CIP are crucial but complicated by its broad onset window and clinical and radiological heterogeneity. The imaging features of CIP frequently overlap with those of infectious pneumonia. Since the emergence of the COVID-19 pandemic, this diagnostic challenge has become even more significant. Sumeet Hindocha et al (164) developed a rigorously validated machine learning tool capable of differentiating CIP from radiation pneumonitis (RP), COVID-19, and other infectious pneumonia. Compared with radiologists, the models for distinguishing RP from COVID-19, CIP from COVID-19, and CIP from non-COVID-19 interstitial pneumonia (IP) demonstrated superior performance, as evidenced by test set AUCs of 0.92 versus 0.8 and 0.8; 0.68 versus 0.43 and 0.4; and 0.71 versus 0.55 and 0.63, respectively.

Recent research has expanded the application scope of radiomics in the treatment of CIP and improved its value in immunotherapy monitoring. The application of radiomics in the treatment of CIP has gradually expanded from initial risk stratification to automatic identification, differential diagnosis, and prognosis prediction.

## 6 Management of CIP

On the basis of the Toxicity Management Guidelines for Immune Checkpoint Inhibitor Pneumonitis (CIP) published by the National Comprehensive Cancer Network (NCCN) (165), CIP is categorized into four grades: Grade 1, asymptomatic, lesions are limited to a single lobe or < 25% of the lung parenchyma; Grade 2, new symptoms or worsening of existing symptoms, including shortness of breath, cough, fever, chest pain, and hypoxia; lesions involve multiple lobes or 25% to 50% of the lung parenchyma, affecting daily life, requiring pharmacological intervention; Grade 3, severe new symptoms, lesions involve all lobes or >50% of the lung parenchyma, self-care ability is limited, oxygen is needed, hospitalization is needed for treatment; and Grade 4, life-threatening respiratory distress syndrome, acute respiratory distress syndrome. Current guidelines recommend the management of CIP on the basis of severity grading (166).

### 6.1 Graded treatment of CIP

For Grade 1 CIP, ICI discontinuation is advised with self-monitoring of symptoms and weekly physical examination and oxygen saturation monitoring during the first 3 weeks. If symptoms occur, chest CT should be implemented in advance. Without clinical progress, a follow-up chest CT after 3–4 weeks should be performed. If imaging shows improvement, resumption of ICI therapy with close follow-up is recommended. In cases of no radiographic improvement, treatment should be escalated to Grade 2, and ICI therapy should be paused.

The recommended approaches for Grade 2 CIP include baseline assessment, such as blood tests (complete blood count, liver and kidney function, electrolytes), and pulmonary function analysis. ICI therapy should be paused until the condition improves to Grade 1 or lower. Intravenous methylprednisolone at 1–2 mg/(kg·d) is advised for 48–72 hours. If symptoms improve, the steroid dose should be tapered over 4–6 weeks and reduced by 5–10 mg weekly. If there is no improvement, treatment should be escalated to Grade 3–4 protocols. If infection cannot be ruled out, empirical antibiotics should be considered. Bronchoscopy with bronchoalveolar lavage is recommended for atypical lesions.

Generally, for Grade 3–4 CIP, ICI therapy should be permanently discontinued. Intravenous methylprednisolone at 2 mg/(kg·d) is recommended. If clinical improvement is observed within 48 hours, continue steroid therapy until symptoms improve to Grade 1 or lower and then taper over 4–6 weeks. Bronchoscopy with bronchoalveolar lavage is advised for atypical lesions, and biopsy may be considered. Empirical antibiotic therapy should be initiated if infection cannot be ruled out. If there is no significant improvement with steroids, alternative therapies such as tocilizumab (8 mg/kg, repeatable after 14 days), infliximab (5 mg/kg, repeatable after 14 days), mycophenolate mofetil (1–1.5 g twice daily), or intravenous immunoglobulin (IVIG) should be considered.

Antimicrobial agents constitute a critical component of the pharmacological management of CIP. Patients who have received antitumor immunotherapy, chemotherapy, radiotherapy, as well as steroid hormones and immunosuppressive therapies for CIP are frequently immunocompromised, rendering them highly susceptible to a range of infectious diseases, including opportunistic infections. Respiratory infections can potentially trigger or worsen CIP, and a subset of patients may concurrently present with both CIP and pulmonary infectious diseases, thereby complicating the clinical management of CIP. In cases of coexisting infection, an individualized antibiotic regimen should be formulated on the basis of the clinical and radiological characteristics of CIP, etiological findings, and the patient’s baseline therapeutic strategy (140). Some experts propose that during the treatment of CIP with steroid hormones and/or immunosuppressants, prophylactic sulfonamide therapy can be administered. In the absence of contraindications, a prophylactic dose of sulfonamide should be routinely administered orally. Common high-risk factors are poorly controlled diabetes mellitus, long-term use of hormones (prednisone equivalent ≥ 20 mg/d) and/or immunosuppressants, bone marrow suppression following oncological treatment, a history of *Pneumocystis jirovecii* infection, and a peripheral blood CD4^+^ T-cell count of less than 400 cells/μl (167–170).

### 6.2 Corticosteroid therapy and escalation strategies for CIP

Corticosteroids are generally recommended as the initial treatment for symptomatic checkpoint inhibitor pneumonia (CIP) patients. Retrospective data indicate that over 90% of Grade 1–2 CPI events can improve or resolve with either the cessation of the drug or the use of corticosteroids, with or without additional interventions. Furthermore, 60%-86% of patients who experience Grade 3 or more severe CIP can achieve remission or cure through corticosteroids or supplementary immunosuppressive therapy (11, 153). Steroid-refractory CIP is defined as the absence of clinical improvement following high-dose corticosteroids administered for 48 hours, which necessitates the introduction of additional immunosuppressive treatments (131).

#### 6.2.1 The occurrence and prognosis of steroid-refractory CIP

According to reported data from relatively large cohorts of CIP patients, the proportion of patients with steroid-refractory CIP ranged from 11.6% (11) to 18.5% (131). KL-6 may serve as a viable screening biomarker for CIP, particularly in NSCLC patients, and could predict steroid responsiveness (171). Immune toxicity has been associated with improved efficacy of immunotherapies. In an advanced NSCLC cohort (171), patients with severe CIP presented the highest overall response rate (ORR) to immune checkpoint inhibitors (ICIs), followed by those with grade 1–2 CIP, compared with non-CIP patients (44.44%, 35.3%, and 28.35%, respectively). However, the prognosis for patients with steroid-refractory CIP is suboptimal. A retrospective cohort study involving 26 patients with steroid-refractory CIP reported a mortality rate of 23% due to pneumonitis and 12% attributed to infections, which may have been associated with immunosuppression (172). Furthermore, the mortality attributable to CIP and/or associated infectious complications was 75% (8/12) in another cohort (131). Compared with steroid-responsive CIP, steroid-refractory CIP is associated with a higher 1-year mortality rate, with a hazard ratio (HR) of 15.1 (95% CI: 3.9–57.8, P < 0.0001) (173).

#### 6.2.2 Advances in the treatment of steroid-refractory CIP

For steroid-refractory CIP, there is currently no unified standardized treatment strategy. Several immunosuppressants, including infliximab, mycophenolate mofetil, intravenous immunoglobulin (IVIG), and cyclophosphamide, are recommended primarily on the basis of their efficacy in treating other steroid-refractory immune-related adverse events (irAEs). However, the efficacy and safety data for these therapies specifically targeting CIP are limited and exhibit significant variability across retrospective studies. Jason Beattie and colleagues reported that among 26 patients receiving additional immunomodulators (tumor necrosis factor-alpha inhibitors and/or mycophenolate) for steroid-refractory CIP, 38% (10/26) achieved durable improvement (172). In another study, Aanika Balaji and colleagues examined 65 patients with CIP, of whom 12 were steroid refractory. Among these patients, those treated with infliximab, with or without IVIG, experienced 100% mortality (5/5 patients), whereas those treated with IVIG alone had a mortality rate of 42.9% (3/7 patients) (131). An earlier study reported improvement in CIP with infliximab in 4 out of 9 patients within a small, retrospective cohort, where bronchoscopy was performed in 67% of cases (172). Given the challenges in differentiating infection from pneumonitis, particularly in severe cases, the mixed efficacy of the aforementioned therapies may be partially attributed to variations in the utility of bronchoscopy.

Agents targeting IL-6R, such as tocilizumab, have been shown to be effective approaches for treating several types of steroid-refractory immune-related adverse events (irAEs) (91), including one case of CIP (174). Clinical trials are currently ongoing to evaluate the safety and efficacy of tocilizumab in combination with ICIs (NCT04940299, NCT03999749). Other reported therapies for steroid-refractory CIP patients include pulse corticosteroids (175), cyclophosphamide (176), and cyclosporine (177).

Given the high mortality associated with severe CIP and the lack of reliable treatments for steroid-refractory cases, there is a pressing need to explore various treatment approaches for these patients in prospective studies. Conducting such trials may be challenging owing to the relatively low incidence of this condition (NCT04438382). Currently, prospective studies are evaluating the role of concurrent immunosuppressants with ICIs in preventing irAEs (NCT04940299, NCT03999749). Notably, the addition of early short-course corticosteroids to ICI therapy has been shown to reduce the incidence of irAEs that lead to ICI discontinuation in patients with NSCLC (5.8% vs. 15.7%, OR = 0.34; 95% CI, 0.12–0.85; p = 0.013) (178). For patients requiring long-term corticosteroid therapy to manage irAEs, prophylaxis against certain opportunistic infections is advisable, even though the incidence of *Pneumocystis jirovecii* pneumonia (PJP) has been reported to be low (169).

### 6.3 Advances in the treatment of chronic CIP

Some researchers propose dividing the clinical phenotypes of CIP into acute, subacute, and chronic phases and dividing the 2 pathological processes of CIP into inflammatory, profibrotic, and fibrotic stages (179, 180). Early-onset CIP often occurs within 6 weeks of patients receiving ICI treatment, and the symptoms are generally severe with a poor prognosis. Late-onset CIP typically appears after 6 weeks of ICI treatment, with fewer symptoms and a better prognosis. Chronic CIP is defined as CIP that persists or worsens after the reduction of steroid treatment, and such patients require more than 12 weeks of immunosuppressive therapy after the discontinuation of ICIs (181). During the chronic CIP phase, fibrotic interstitial lung diseases (such as complete damage to normal lung structure, thickening and obstruction of blood vessels) are histologically dominant, which may impair the efficiency of blood–gas exchange in the lungs (182, 183). Typical sequelae may include persistent pulmonary interstitial fibrosis and reduced pulmonary function caused by severe CIP (54). These concepts provide new directions for the treatment of CIP beyond immunosuppressive therapy, namely, antifibrotic treatment. Anti-fibrosis agents such as pirfenidone have demonstrated efficacy (184). In a retrospective study (185), compared with the glucocorticoid-only group, the glucocorticoid-pirfenidone group demonstrated significant improvements in forced vital capacity (FVC), diffusing capacity of the lung for carbon monoxide (DLCO), 6-minute walk distance (6MWD), and oxygen saturation without supplemental oxygen (P < 0.05). Among patients with grade 2 immune checkpoint inhibitor-related pneumonia (CIP), those receiving combination therapy exhibited a significantly shorter duration of symptom improvement than those treated with glucocorticoids alone did. Furthermore, following the resumption of immunotherapy, the incidence of CIP recurrence was lower in the glucocorticoid-pirfenidone group than in the glucocorticoid-only group. Nintedanib is a small molecule tyrosine kinase inhibitor that has been approved for the antifibrotic treatment of patients with idiopathic pulmonary fibrosis (IPF) and chronic interstitial lung disease (ILD) (186). Additionally, nintedanib has demonstrated efficacy against solid tumors in multiple clinical trials. For patients with advanced squamous carcinoma, the LUME-Lung 3 study reported that with first-line combination treatment of nintedanib plus cisplatin plus gemcitabine, the disease control rate (DCR) was 81.3%, the median progression-free survival (mPFS) was 4.2 months, and the median overall survival (mOS) was 6.7 months (187). In lung adenocarcinoma, the combination treatment of nintedanib and docetaxel has been approved by the European Union as a first-line chemotherapy regimen (https://www.ema.europa.eu/en/medicines/human/EPAR/vargatef). Several case reports have documented the use of nintedanib for salvage treatment in refractory and chronic CIP patients, with clinical efficacy (186, 188).

### 6.4 ICI rechallenge in resolved CIP: strategies and long-term vigilance

When grade 1–2 CIP events resolve or revert to grade 1, patients may continue their previous ICI regimen. The recurrence of CIP should be closely monitored, particularly for early-onset irAEs (189). Delaunay et al. reported that among 10 patients with non-small cell lung cancer (NSCLC) who were rechallenged with ICIs after initially managing grade 1–2 CIP with ICI withdrawal or corticosteroids, 3 patients (30%) experienced recurrent interstitial lung disease (ILD) (153). A larger retrospective cohort study revealed that 9 patients (20.0%) developed recurrent pneumonitis, and 11 patients (24.4%) experienced new irAEs among 45 CIP patients who underwent ICI rechallenge (190). A more recent analysis (191) revealed that while ICI rechallenge beyond disease progression did not introduce new safety concerns, it did not improve clinical outcomes in patients with locally advanced or metastatic NSCLC. However, patients who demonstrate a favorable response to initial ICI treatment still benefit from subsequent ICI rechallenge therapy. Similarly, systematic analysis revealed that the incidence rates of all-grade and high-grade (grade 3 or 4) irAEs were not significantly different between initial and readministered ICIs, and no significant difference in overall survival (OS) was observed between the ICI rechallenge and discontinuation cohorts (192). For patients who have recovered from grade 2 pneumonitis, cautious reintroduction of ICIs is advised, particularly for those with a positive response to initial ICIs and without feasible alternative therapies. This resumption may be accompanied by concomitant prednisone treatment, typically at a dosage of 20–30 mg/day, and should be conducted under close monitoring with chest CT (189). Although reintroduction of ICIs has been proven safe in rare and carefully selected cases (193), it is not recommended for patients who have recovered from grade 3 CIP events. For patients with grade 4 CIP, the use of ICIs should be permanently avoided.

Overall, most cases of CIP can be effectively managed through the withholding of ICIs and the administration of corticosteroids. Although severe cases are relatively uncommon, they are associated with poor prognosis and high mortality, underscoring the need for prompt recognition and intervention. Currently, there are no prospective trials evaluating the efficacy of treatments for severe cases; thus, no universal guidelines for optimal therapy beyond corticosteroids exist. For patients who have recovered from CIPs, immunotherapy rechallenge may be feasible, particularly for those who experienced low-grade CIPs and benefit from initial ICI treatment; however, the recurrence of CIP or the emergence of new irAEs should be closely monitored.

## 7 Conclusion and future points

With the expanding application of immune checkpoint inhibitors (ICIs) in oncology, the real-world incidence of immune checkpoint inhibitor-related pneumonia (CIP) has been progressively increasing. Despite advances in understanding its pathogenesis, imaging features, and therapeutic strategies, the prediction and early diagnosis of CIP remain challenging in clinical practice. The lack of specific imaging patterns and validated biomarkers often leads to delayed diagnosis and treatment, particularly contributing to higher mortality rates among patients with steroid-refractory pneumonia. Emerging studies investigating the underlying mechanisms and potential predictive biomarkers of CIP have identified associations between its occurrence, severity, and response to steroid therapy and various inflammatory mediators, chemokines, autoantibodies, and certain genetic predispositions. Additionally, evidence suggests that the baseline neutrophil-to-lymphocyte ratio (NLR) and platelet-to-lymphocyte ratio (PLR) are correlated with the risk of immune-related adverse events (irAEs) in patients with solid tumors receiving ICIs (147), whereas the baseline lymphocyte and eosinophil counts have also been linked to irAE development (194). Nevertheless, these biomarkers have not yet been widely adopted in clinical settings, and their diagnostic sensitivity and specificity require further validation through large-scale prospective cohort studies.

Radiomics and machine learning, as critical branches of artificial intelligence in medical imaging, enable the extraction of many quantitative features from high-resolution CT scans. These technologies support the clinical risk stratification of immune checkpoint inhibitor-related pneumonia (CIP) and facilitate its differential diagnosis from other types of pneumonia, such as radiation pneumonitis and infectious pneumonia. Recently, various convolutional neural network (CNN)-based deep learning models and multimodal models that integrate clinical, imaging, and pathological data have demonstrated outstanding classification performance, with area under the curve (AUC) values generally exceeding 0.9 (158, 164), significantly surpassing traditional radiological evaluation methods. For example, Wang et al. utilized deep learning visualization techniques and reported that regions beyond the tumor—such as pleural retraction, pleural effusion, and vascular abnormalities—are crucial for predicting the risk of CIP during immunotherapy (195).

Despite their promising applications in CIP diagnosis and risk prediction, several challenges remain: a lack of standardized protocols for image acquisition; insufficient repeatability and stability in image segmentation, leading to variability in region of interest (ROI) delineation across different software platforms, drawing methods, and observers; and most current studies on CIP are single-center retrospective analyses with limited sample sizes. To validate these findings more robustly, large-scale multicenter prospective studies along with external dataset validation and training are needed. To date, research on CIP has focused primarily on predictive modeling and differential diagnosis, with relatively limited exploration of disease prognosis and therapeutic response. This gap highlights an important direction for future investigations. In model development, the acquisition of high-quality images is fundamental to ensuring model robustness. During radiomic feature extraction, standardization of the slice thickness, imaging protocols, and ROI delineation procedures is essential. Integrating radiomic features with patients’ clinical profiles, cytokine levels, autoantibody panels, and genetic predispositions, followed by advanced bioinformatics analysis, may yield models with enhanced accuracy in diagnosis, prognosis, and outcome prediction (196).

In conclusion, the prediction, diagnosis, and management of immune checkpoint inhibitor-related pneumonitis (CIP) are progressively advancing toward the integration of multiomics biomarkers and imaging artificial intelligence technologies. Future efforts should focus on strengthening interdisciplinary collaboration, validating the clinical utility of novel models through prospective, multicenter studies, and facilitating the broad implementation of these cutting-edge tools in routine clinical practice. Only through such systematic approaches can the mortality and treatment discontinuation risks associated with CIP be effectively mitigated, thereby fully harnessing the therapeutic potential of immunotherapy.
